# Renal Kallikrein Activation and Renoprotection after Dual Blockade of Renin-Angiotensin System in Diet-Induced Diabetic Nephropathy

**DOI:** 10.1155/2015/310645

**Published:** 2015-03-30

**Authors:** Xia Zou, Xiao-xi Zhang, Xin-yu Liu, Rong Li, Min Wang, Wei-jie Wu, Yi Sui, Hai-lu Zhao

**Affiliations:** ^1^Center for Diabetic Systems Medicine, Guangxi Key Laboratory of Excellence, Guilin Medical University, Guilin 541004, China; ^2^Molecular Endocrinology and Toxicology Laboratory, Department of Biology, Hong Kong Baptist University, Kowloon 999077, Hong Kong; ^3^Department of Endocrinology, Guangdong Hospital of Traditional Chinese Medicine, Guangzhou 510120, China

## Abstract

*Purpose*. The objective of this study is to investigate the effect of dual blockage of renin-angiotensin system (RAS) on renal kallikrein expression and inflammatory response in diabetic nephropathy (DN). *Methods*. Rats were randomly divided into 5 groups with 10 rats in each group: normal control; DN model induced by high fat and high sucrose diets; and DN treated with either benazepril 10 mg/kg/d, irbesartan 30 mg/kg/d, or both. After 8-week treatment, we examined changes in the kidney histopathology, function and immunohistochemical stain of kallikrein, macrophage marker CD68, and profibrotic markers transforming growth factor- (TGF-) *β* and *α*-smooth muscle action (SMA). *Results*. DN rats showed enlarged kidneys with glomerulosclerosis, interstitial chronic inflammation and fibrosis, and proteinuria. All the pathological damage and functional impairments were improved after the RAS blockades (all *P* < 0.05). Compared with monotherapy, combined treatment further alleviated the kidney impairments in parallel to increased tubular immunoreactivity for kallikrein and decreased immunopositive cells for CD68, TGF-*β*, and *α*-SMA. *Conclusion*. The renoprotective effects of the dual RAS blockade in diabetic nephropathy may be attributed to improved tubular kallikrein expression and interstitial inflammatory response.

## 1. Introduction

Diabetic nephropathy (DN) is one of the most common microvascular complications of diabetes mellitus (DM) and a leading cause of end-stage renal disease (ESRD) [1]. China currently has approximately 100 million adults with type-2 diabetes and 150 million people with prediabetes [2, 3]. Alarmingly, 120 million Chinese adults may suffer from chronic kidney disease manifested predominantly as albuminuria [4]. Albuminuria is the major determinant of DN and an important risk factor for cardiovascular disease in patients with diabetes. Indeed, hospitalized Chinese patients with type-2 diabetes showed 44% of microalbuminuria, 12% of macroalbuminuria, and 17% of cardiovascular disease [5]. Therefore, type-2 diabetes and chronic kidney disease have become major public health problems in China. These chronic noncommunicable diseases are mutually interrelated with unmet medical needs [6].

Renin-angiotensin system (RAS) plays a key role in the pathogenesis of diabetic nephropathy. RAS blockage including angiotensin-converting enzyme inhibitors (ACEI) and angiotensin receptor blockers (ARB) is currently used for DN treatment [7, 8]. Furthermore, DN prevention also relies substantially on the prescription of ACEI and ARB in clinical settings [9]. ACEI such as benazepril have beneficial effects on DN independent of blood pressure-lowering effect and plasma angiotensin II levels [10]. Similarly, ARB such as irbesartan also reveal renoprotective effects in landmark clinical trials [11–14]. In this regard, researches of dual blockade with combined ACEI and ARB are ongoing to maximize the beneficial effects and minimize the adverse events.

The disruption of the RAS pathway leads to negative feedback and compensatory kallikrein system activation [15–17]. Abnormal activation of kallikrein excitation peptide system (KKS) mainly induces DN progression [10]. Studies have found that heart tissue kallikrein activity significantly increases the effect of ACEI in type-2 diabetes [18], indicating mechanistic interaction between KKS and RAS [19].

The aim of the present study was to investigate the effect of combined intervention with ACEI-benazepril and ARB-irbesartan on experimental DN rats. We hypothesized that the combined therapy could protect against hyperglycemia-induced chronic renal impairments through renal tissue kallikrein system activation and inflammatory pathway inhibition.

## 2. Materials and Methods

### 2.1. Animals and Diets

Male Sprague Dawley rats (230 ± 20 g body weight) were purchased from Guilin Medical Laboratory Animal Center. The animals were housed in a specific pathogen-free (SPF) laminar flow cabinet. Temperature and humidity were maintained at 25 ± 1°C and 50 ± 5.0%, respectively. The rats, which were exposed to light, were regulated in 12 : 12 h light-dark cycle. The diet and sterilized water were used to feed these rats.

Ethical approval for animal studies was according to the Animal Experimentation Ethics Committee of Guilin Medical University. In this study, we used high fat and high sucrose diets (HFD) and conventional diet (5001 Rodent Diet; LabDiet, St. Louis, MO, USA). HFD contains 10% lard, 20% sucrose, 2% egg yolk powder, 3% cholesterol, and 65% conventional diet.

### 2.2. Diet-Induced Diabetic Nephropathy and RAS Blockade

After adaptive feeding for one week, the rats were fed with conventional diet (*n* = 10) or HFD (*n* = 50) for 4 weeks. Then, the HFD-fed rats received intraperitoneal injection with low dose (35 mg/kg) of streptozotocin (Sigma, St. Louis, MO, USA) to hasten diabetic status. Streptozotocin was freshly prepared by dissolving in sterile sodium citrate-trisodium citrate buffer (0.1 mmol/L, PH 4.2). Meanwhile, the rats fed with conventional diet also received intraperitoneal injection of the same volume of the buffer. Six weeks later, DN rats were defined by the following criteria: (1) blood glucose level ⩾16.7 mmol/L; (2) 1.5-fold increase in urine volume compared to the baseline; and (3) urinary albumin excretion rate beyond 30 mg/24 h [20, 21]. A total of 40 HFD-fed rats met the criteria and eventually were included in this study.

Subsequently, the rats were divided into 5 groups and each group had 10 rats: (1) normal control; (2) DN model control; (3) DN rats treated with benazepril 10 mg/kg/d; (4) DN rats treated with irbesartan 30 mg/kg/d; and (5) DN rats treated with both benazepril 10 mg/kg/d and irbesartan 30 mg/kg/d. Blood glucose level was determined via taking blood from tail vein once a week, using the Precision Xtra blood glucose monitoring system (Alameda, CA, USA).

At the end of 8-week treatments, 24 h urine from each group was collected using metal metabolism cage to calculate total urine volume and measure albumin excretion rate. Fasting serum insulin concentrations were measured using enzyme immunoassay and rat insulin ELISA kit (Mercodia, Uppsala, Sweden). HOMA-IR (homeostasis model assessment of insulin resistance) index was estimated with the formula HOMA-IR = fasting serum insulin (mU/L) × fasting serum glucose (mmol/L)/22.5 [22]. Kidney index here reflects kidney hypertrophy estimated by the kidney weight/body weight (mg/g).

### 2.3. Immunohistochemistry, Light, and Fluorescence Microscopy

All the kidney tissue samples were fixed in 10% buffered formalin and embedded in paraffin. Serial sections (4 *μ*m) were cut perpendicular to the longest axis of the kidney. Sections were stained by primary antibodies including kallikrein 1, the phagocytic immunomarker CD68 (macrophage) [23], the proinflammatory and profibrotic markers transforming growth factor- (TGF-) *β*1, and *α*-smooth muscle action (SMA) (Santa Cruz, CA, USA), as described in our prior reports [22–24]. Stained slides were counterstained with either DAB (brown) for light microscopy or antifading DAPI (blue) for immunofluorescence microscopy, followed by examination with a Zeiss A2 imaging microscope (Carl Zeiss, Hamburg, Germany). When checking the stained slides, the pathologist was blinded for animal grouping.

### 2.4. Statistical Analyses

All data were analyzed using the SPSS 19.0 software. Comparisons among the five groups were analyzed by one-way analysis of variance (ANOVA), followed by Tukey* post hoc* multiple comparison test. Two-tailed *P* values less than 0.05 were considered statistically significant. All data were presented as mean ± SD.

## 3. Results

### 3.1. Characterization of the Diet-Induced Diabetic Nephropathy

Before injecting streptozotocin, the average concentrations of blood glucose between normal control and HFD-fed groups were not significantly different. However, the HFD stimulated a significant increase in body weight, fasting serum insulin, and HOMA-IR (Table 1).

Six weeks after injecting the low dose of streptozotocin, HFD-fed rats (*n* = 40) showed hyperglycemia with average blood glucose level over 16.7 mmol/L, polyuria with 1.5-fold increased urine volume, and albuminuria with urinary albumin excretion rate higher than 30 mg/24 h. All the biochemical outcomes indicate establishment of DN model by HFD plus low dose streptozotocin.

### 3.2. Renoprotection by RAS Blockade

**Figure 1:**
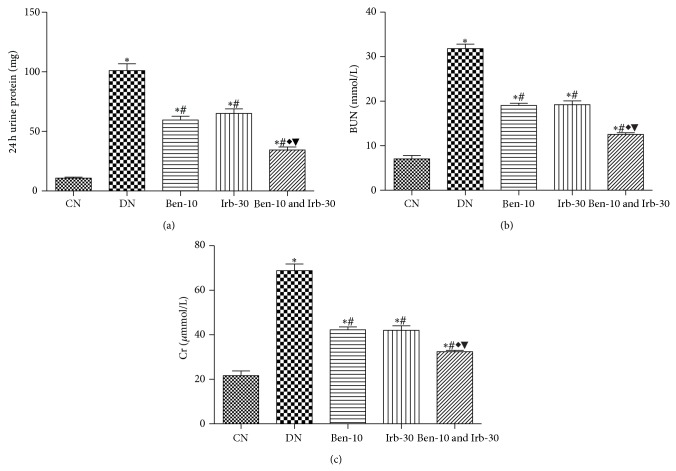
The effect of benazepril and irbesartan on the levels of 24 h urine protein (a), BUN (b), and Cr (c); CN: normal control group, group 1; DN: diabetic nephropathy control group, group 2; Ben-10, group 3 (benazepril 10 mg/kg/d); Irb-30, group 4 (irbesartan 30 mg/kg/d); and Ben-10 and Irb-30, group 5; and compared with group 1, ^∗^ *P* < 0.05; compared with group 2, ^#^ *P* < 0.05; compared with group 3, ^◆^ *P* < 0.05; compared with group 4, ^▼^ *P* < 0.05; BUN: blood urea nitrogen; Cr: serum creatinine.

**Figure 2:**
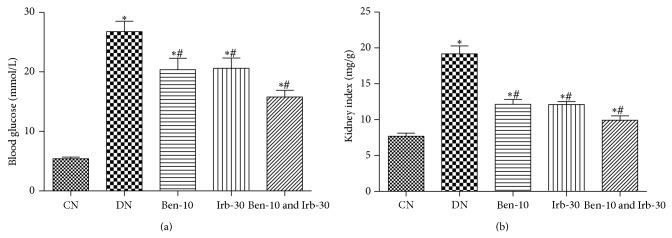
The effect of benazepril and irbesartan on the levels of blood glucose (a) and kidney index (b); CN: normal control group, group 1; DN: diabetic nephropathy control group, group 2; Ben-10, group 3 (benazepril 10 mg/kg/d); Irb-30, group 4 (irbesartan 30 mg/kg/d); and Ben-10 and Irb-30, group 5; and compared with group 1, ^∗^ *P* < 0.05; compared with group 2, ^#^ *P* < 0.05.

To evaluate renal function changes after RAS blockade, 24 h urine protein, blood urea nitrogen, and serum creatinine were determined. Compared with the normal controls, the DN rats showed kidney dysfunction echoed by elevated serum levels of 24 h urine protein (Figure 1(a)) and blood urea nitrogen (Figure 1(b)) and serum creatinine (Figure 1(c)) (all *P* < 0.05). The renal dysfunction significantly improved after the treatments with ACEI, ARB, or both (Figure 1). Notably, the dual RAS blockade almost normalized the renal functions. Similar renoprotective effects were observed between the ACEI and ARB treatments (Figure 2(a)). Interestingly, obvious blood glucose lowering was achieved by the dual RAS blockade versus DN control and monotherapy.

Pathological changes in the DN rats included kidney hypertrophy reflected by the increased kidney index (Figure 2(b)), mild-to-moderate glomerulosclerosis, tubular atrophy with basement membrane thickening, arteriosclerosis, and interstitial fibrosis with chronic inflammatory infiltration (data not shown). Treatments with ACEI, ARB, or both generally ameliorated the pathological alterations. Consistent with the renal function improvement, pathological correction by the combined therapy was superior to any monotherapy.

### 3.3. Renal Immunoreactivity of Kallikrein

**Figure 3:**
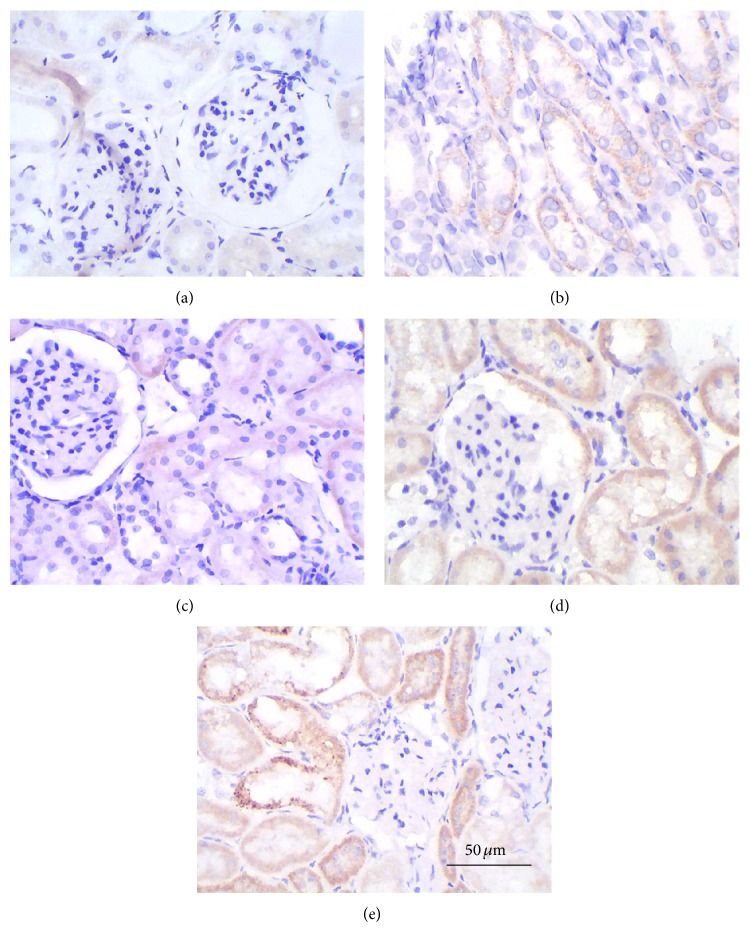
The immunohistochemical staining of kidney (HE 400x) in five groups. (a) Normal control group; (b) diabetic nephropathy control group; (c) benazepril (10 mg/kg/d); (d) irbesartan (30 mg/kg/d); and (e) benazepril (10 mg/kg/d) and irbesartan (30 mg/kg/d); scale bar, 50 *μ*m.

Figure 3 shows the immunoreactivity of kallikrein predominantly localized in renal tubular cells and generally negative in glomeruli. The DN control rats showed very fewer cells reactive for kallikrein (Figure 3). Tubular cells positive for kallikrein substantially increased in rats which received the ACEI, ARB, or both (Table 2). The dual blockade was superior to monotherapy for stimulating kallikrein immunoreactivity.

### 3.4. Inflammatory CD68-Immunoreactive Cells

**Figure 4:**
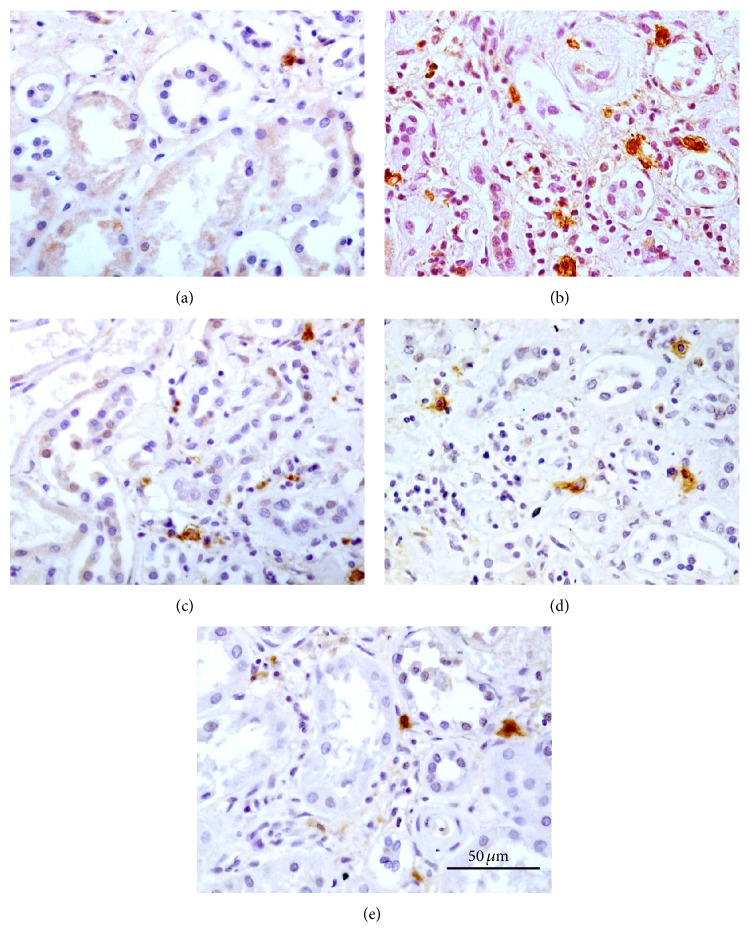
Light microscopy of immunohistochemical stain with CD68. Kidney tissue sections (4 *μ*m) were stained with the phagocytic marker CD68 (brown) and examined under a Zeiss A2 imaging microscope (Carl Zeiss, Hamburg, Germany). Immunoreactive cells are mainly interstitial large macrophages intermixed with chronic inflammatory infiltrates. (a) Normal control (CN); (b) model of diabetic nephropathy that never had any treatment (DN); (c) treatment with benazepril 10 mg/kg/d (Ben); (d) treatment with irbesartan 30 mg/kg/d (Irb); (e) combined treatment with benazepril 10 mg/kg/d and irbesartan 30 mg/kg/d (Ben and Irb). Original magnification, ×200; scale bar, 50 *μ*m.

Cells immunoreactive for the inflammatory marker CD68 were mainly interstitial large macrophages intermixed with chronic inflammatory infiltrates (Figure 4). The CD68-positive cells were rare in normal control rats (Figure 4(a)) but frequently seen as diffuse infiltrates in the DN rats (Figure 4(b)). Treatments with the RAS blockers prevented the chronic inflammatory infiltrates reactive for the phagocytic marker CD68. The dual blockade was better than the ACEI (Figure 4(c)) and ARB (Figure 4(d)) to diminish the CD68-stained cell infiltration (Figure 4(e)). In general, the density of CD68-positive cells was in parallel to the severity of structural damage including glomerulosclerosis, arteriosclerosis, and interstitial fibrosis.

### 3.5. Inflammatory Transforming Growth Factor-*β* and *α*-Smooth Muscle Action

**Figure 5:**
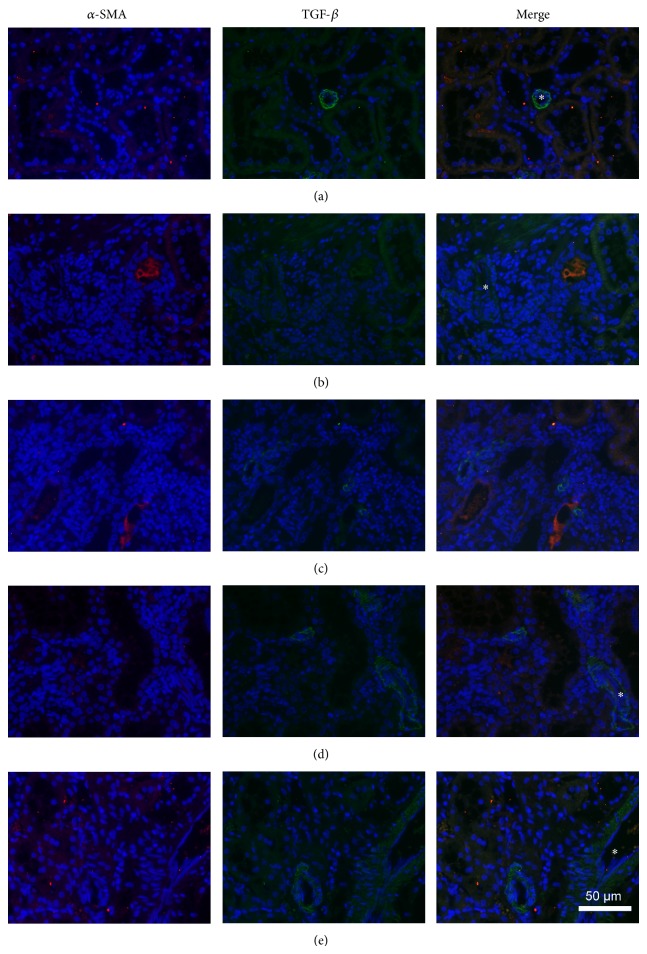
Immunofluorescence microscopy of *α*-smooth muscle action and transforming growth factor-*β*. Kidney tissue sections (4 *μ*m) were stained with the activated fibroblast marker *α*-smooth muscle action (green) and the profibrotic cytokine transforming growth factor-*β* (red) and examined under a Zeiss A2 imaging microscope (Carl Zeiss, Hamburg, Germany). Both interstitial fibroblasts and vascular smooth muscle cells are positive for *α*-smooth muscle action (green). Consistent with the immunostain of the phagocytic marker CD68 (Figure 4), inflammatory cells immunoreactive for transforming growth factor-*β* (red) are predominantly interstitial large macrophages and frequently intermixed with infiltrating lymphocytes. (a) Normal control (CN); (b) model of diabetic nephropathy that never had any treatment (DN); (c) treatment with benazepril 10 mg/kg/d (Ben); (d) treatment with irbesartan 30 mg/kg/d (Irb); (e) combined treatment with benazepril 10 mg/kg/d and irbesartan 30 mg/kg/d (Ben and Irb). Original magnification, ×200; scale bar, 50 *μ*m.

In general, chronic inflammatory cells immunoreactive for the profibrotic marker TGF-*β*1 were interstitial macrophages frequently intermixed with infiltrating lymphocytes (Figure 5), consistent with the CD68 reactivity in Figure 4. In contrast, both interstitial fibroblasts and vascular smooth muscle cells were positive for the activated fibroblast marker *α*-SMA. *α*-SMA positivity localized mainly with arteriosclerosis and interstitial fibrosis whereas immunoreactivity of TGF-*β*1 was accompanied by chronic inflammatory infiltration (Figure 5). Among the five groups of animals, the DN rats showed a few more cells positive for TGF-*β*1 and *α*-SMA than normal controls and rats given RAS blockers (Figure 5). Moreover, combined treatment appeared better than monotherapy in attenuating the immunofluorescence of TGF-*β*1 and *α*-SMA. Finally, the ACEI and ARB demonstrated similar effects on the immunofluorescence.

## 4. Discussion

This study shows tubular kallikrein activation and inflammatory amelioration likely underlying the renoprotective effects of RAS blockades in diet-induced diabetic nephropathy. Dual RAS blockade with both ACEI and ARB may delay the development of diet-induced diabetic nephropathy through compensatory kallikrein upregulation and inflammatory downregulation.

Both kallikrein-kinin system (KKS) and renin-angiotensin system (RAS) have been implicated in the pathogenesis of diabetic nephropathy (DN) [25, 26]. The KKS serves as the physiological and pathophysiological counterbalance to the RAS. Tissue kallikrein 1 is a member of the tissue kallikrein family that is mainly responsible for the generation of kinins, while bradykinin (BK) is the principal kinin responsible for the physiological actions [26]. In the kidney, kallikrein 1 is confined to the tubules in both healthy and diabetic states. Consistent with our findings in this study, a reduction of renal kallikrein excretion has been found in diabetic individuals and patients with diabetic nephropathy [27]. Early clinical evidence has indicated a significantly lower urinary kallikrein excretion in type-2 diabetic patients with nephropathy than in diabetic patients without nephropathy and in control subjects [28]. In experimental type-1 diabetes, kallikrein protects against the development of microalbuminuria [29]. In streptozotocin-induced diabetic rats, kidney tissue level and excretion of active kallikrein were reduced after 3 weeks compared with age-matched nondiabetic control rats, and despite increased kidney size, renal plasma flow was reduced in the diabetic rats [30]. Increased urinary BK levels found in severely hyperglycemic diabetic rats are related to increased filtration of components of the plasma KKS and renal kininogen synthesis in combination with decreased renal kinin-degrading activity [31]. All these findings are similar to the present study. Furthermore, activated KKS under diabetic condition may be beneficial in preventing podocyte loss in diabetic nephropathy [32]. Previous reports have shown that intravenous delivery of the human tissue kallikrein gene reduced blood pressure and plasma insulin levels in fructose-induced hypertensive rats with insulin resistance [33]. Furthermore, a recombinant adenoassociated viral vector expressing the human tissue kallikrein cDNA as a sole, long-term therapy could correct insulin resistance and prevent renal damage in streptozotocin-HFD-induced type-2 diabetic rats [34]. In this study, the intensity of tubular kallikrein expression was related to the severity of diabetic nephropathy defined by polyuria, proteinuria, kidney hypertrophy, and chronic inflammatory infiltration. All these findings suggest that the KKS may serve as a therapeutic target in delaying the progression of diabetic nephropathy [35].

The present study also highlights the close interaction between the KKS and the RAS. Both urinary kallikrein excretion and kallikrein excretion rate (24 h excretion of urinary kallikrein/24 h creatinine clearance) in hypertensive diabetic patients with nephropathy were significantly lower than in normotensive patients with nephropathy [28]. The decrease in urinary kallikrein is parallel to the existence of diabetic nephropathy with arterial hypertension [36]. Although the basal plasma renin activity in patients with type-2 diabetes was not significantly different from controls, both the renin activity and kallikrein levels increased after furosemide in controls while in diabetics this response was severely blunted, indicating early derangement of the KKS and the RAS as renal hemodynamic mechanisms heralding the onset of nephropathy [37]. Moreover, the suppressed renal KKS is evident not only in the whole kidney but in each nephron which is still functioning [38].

Furthermore, prior study has revealed that the reduction of kinin metabolism by ACEI might be involved in the beneficial effects exerted by these compounds in diabetic kidney functions [39]. Although an earlier study questions the postulate of a role of the KKS in the initiation of essential hypertension [40], interaction between the KKS and RAS may partially contribute to the multifactorial nature of diabetic nephropathy.

In addition to kidney haemodynamics modulated by the KKS-RAS counterbalance, inflammatory response appears to change after the initiation of diabetic nephropathy. The present study suggests potential correlation between the kallikrein activation and inflammatory amelioration. A recent experimental study showed the tissue kallikrein mediated proinflammatory pathways and activation of protease-activated receptor-4 in proximal tubular epithelial cells [41]. Our study has several limitations. The kallikrein 1 level was identified by immunohistochemistry with light microscopy. The immunoreactivity of kallikrein 1 was confined in the kidney tubular epithelial cells. Levels of kallikrein 1 protein were not measured by Western blot. Due to lack of blood and urine samples, we could not measure the levels of plasma kallikrein 1, urinary kallikrein excretion, and kallikrein excretion rate (24 h excretion of urinary kallikrein/24 h creatinine clearance). Our future work will explore interactions between the kallikrein activation and inflammatory pathway by detailed measurements of kallikrein and cytokine levels in samples of plasma, kidney tissues, and urine.

Understanding of the RAS has shifted from the classical limited-proteolysis linear cascade to a cascade with multiple mediators, multiple receptors, and multifunctional enzymes. The homologue of ACE, ACE2, is insensitive to ACE inhibitors and forms angiotensin (1-7) [Ang-(1-7)] from angiotensin II (Ang II) with high efficiency. One important receptor for Ang-(1-7) is the Mas. The ACE2/Ang-(1-7)/Mas axis has putative role as an ACE-Ang II-AT1 receptor counterregulatory axis within the RAS [42, 43]. In the present study, the dual RAS blockade exerted significant blood glucose lowering action for renoprotection. A landmark study has shown that the combination of losartan and lisinopril was associated with reduced Ang II formation and elevated cardiac ACE2 activity for increased Ang II metabolism [44]. Moreover, the renal cortex ACE2 formation was upregulated with increased levels of Ang-(1-7) in the urine of animals after ACEI or ARB [45]. These findings provide mechanistic insight of this renoprotective axis of the RAS. Indeed, the ACE2/Ang-(1-7)/Mas axis can inhibit hepatic insulin resistance [46]. Furthermore, Ang-(1-7) generated by proximal tubular ACE2 inhibits Ang II-stimulated MAPK phosphorylation and TGF-*β* expression in proximal tubular cells [47]. Taken together, the ACE2/Ang-(1-7)/Mas axis exerts protective actions in diabetes, chronic kidney disease, hypertension, and other cardiovascular and metabolic disorders [48].

In the present study, we have no intension to promote the combined treatment of dual RAS blockade due to the multifactorial nature of diabetic nephropathy. Furthermore, a new clinical trial also provides evidence that combination therapy with ACEI and ARB is associated with an increased risk of adverse events among patients with diabetic nephropathy [49]. Taken together, new research questions remain for the role of kallikrein as a therapeutic target in diabetic nephropathy [18, 35, 50, 51].

In conclusion, kidney tissue kallikrein activation and inflammatory amelioration may underlie the renoprotective effects by the RAS blockade in diet-induced diabetic nephropathy. Further investigations are required to dissect the mechanistic interactions between the kallikrein-kinin system and renin-angiotensin system.

## Figures and Tables

**Table 1:** Comparison of body weight, fasting blood glucose, blood glucose at 2 hours in OGTT, fasting plasma insulin, and insulin resistance index.

Group	Body weight (g)	Fasting blood glucose (mmol/L)	Blood glucose at 2 hours in OGTT (mmol/L)	Fasting plasma insulin (mU/L)	Insulin resistance index
CN *n* = 10	370.80 ± 14.44	5.62 ± 0.79	5.88 ± 0.97	15.98 ± 1.58	4.01 ± 0.86

HFD *n* = 40	397.67 ± 30.65^*^	5.69 ± 0.61	6.13 ± 0.65	26.32 ± 2.05^*^	6.67 ± 0.98^*^

CN: normal diet control group; HFD: high fat and high sucrose diets group and ^*^ *P* < 0.05 compared with control group; *n*: the number of samples; OGTT: oral glucose tolerance test.

**Table 2:** Expression of kallikrein 1 in renal tissue.

Group	The number of samples	Kallikrein 1 integral optical density value
1	10	9.392 ± 0.597
2	7	21.839 ± 4.686^*^
3	10	30.312 ± 7.700^∗#^
4	9	31.717 ± 5.147^∗#^
5	8	46.119 ± 5.385^∗#◆▼^

Group 1, normal control group; group 2, diabetic nephropathy control group; group 3, benazepril (10 mg/kg/d); group 4, irbesartan (30 mg/kg/d); and group 5, benazepril (10 mg/kg/d) & irbesartan (30 mg/kg/d); and compared with group 1, ^*^ *P* < 0.05; compared with group 2, ^#^ *P* < 0.05; compared with group 3, ^◆^ *P* < 0.05; compared with group 4, ^▼^ *P* < 0.05.
